# Nanoscale inhomogeneity of charge density waves dynamics in La_2−x_Sr_x_NiO_4_

**DOI:** 10.1038/s41598-022-18925-y

**Published:** 2022-09-24

**Authors:** Gaetano Campi, Antonio Bianconi, Boby Joseph, Shrawan Kr Mishra, Leonard Müller, Alexey Zozulya, Agustinus Agung Nugroho, Sujoy Roy, Michael Sprung, Alessandro Ricci

**Affiliations:** 1 Institute of Crystallography, CNR, Via Salaria Km 29.300, 00015 Monterotondo Roma, Italy; 2 Rome International Center Materials Science Superstripes (RICMASS), Via Dei Sabelli 119A, 00185 Roma, Italy; 3 Elettra Sincrotrone Trieste, Strada Statale 14-Km 163.5, AREA Science Park, Basovizza, 34149 Trieste, Italy; 4 School of Materials Science and Technology, Indian Institute of Technology (BHU), Varanasi, 221005 India; 5 Advanced Light Source, Lawrence Berkeley National Laboratory, Berkeley, CA 94720 USA; 6 Deutsches Elektronen-Synchrotron DESY, Notkestr. 85, 22607 Hamburg, Germany; 7 Faculty of Mathematics and Natural Sciences, Institut Teknologi Bandung, Jl. Ganesha 10, Bandung, 40132 Indonesia; 8 Present Address: European XFEL, Holzkoppel 4, 22869 Schenefeld, Germany; 9 Present Address: Duferco Corporate Innovation, Via Trevano 2A, 6900 Lugano, Switzerland

**Keywords:** Materials science, Condensed-matter physics, Superconducting properties and materials, Physics, Chemical physics

## Abstract

While stripe phases with broken rotational symmetry of charge density are known to emerge in doped strongly correlated perovskites, the dynamics and heterogeneity of spatial ordering remain elusive. Here we shed light on the temperature dependent lattice motion and the spatial nanoscale phase separation of charge density wave order in the archetypal striped phase in La_2−x_Sr_x_NiO_4+y_ (LSNO) perovskite using X-ray photon correlation spectroscopy (XPCS) joint with scanning micro X-ray diffraction (SµXRD). While it is known that the CDW in 1/8 doped cuprates shows a remarkable stability we report the CDW motion dynamics by XPCS in nickelates with an anomalous quantum glass regime at low temperature, T < 65 K, and the expected thermal melting at higher temperature 65 < T < 120 K. The nanoscale CDW puddles with a shorter correlation length are more mobile than CDW puddles with a longer correlation length. The direct imaging of nanoscale spatial inhomogeneity of CDW by scanning micro X-ray diffraction (SµXRD) shows a nanoscale landscape of percolating short range dynamic CDW puddles competing with large quasi-static CDW puddles giving rise to a novel form of nanoscale phase separation of the incommensurate stripes order landscape.

## Introduction

Inhomogeneity of charge density waves (CDW) in doped perovskites based on copper^1–14^ and nickel^15–35^ is today of high increasing interest for understanding the intrinsic disorder in quantum complex matter^36–49^ which control emerging quantum functionalities (e.g. high temperature superconductivity, metal–insulator transitions). Novel experimental methods show that nanoscale phase separation in charge, orbital, spin, and lattice degrees of freedom at multiple length scales gives rise to rich landscapes in quantum complex materials. Nowadays, scanning techniques using synchrotron X-ray beams focused down to the nanometer scale have allowed us to visualize unique spatial complexity in quantum materials^41–49^. As relevant examples, we mention here the imaging of intrinsic phase separation in high temperature cuprate superconductors forming a network of nanoscale oxygen rich patches, interspersed with oxygen depleted regions in HgBa_2_CuO_4+y_^10^, La_2_CuO_4+y_^44,45^ and YBa_2_Cu_3_O_6+y_^47,48^. Such an intrinsic phase separation has been observed for iron-based chalcogenide superconductors A_x_Fe_2−y_Se_2_ and Sc doped Mg_1−x_Sc_x_B_2_^43^, too. Moreover, scanning micro X-ray diffraction (SµXRD) has succeeded to visualize the nanoscopic phase separation with the formation of Spin Density Waves (SDW) puddles^28^.

Here, we focus our studies on La_2−x_Sr_x_NiO_4_, a 2D doped Mott insulator, which is isostructural with the superconducting cuprate La_2−x_Sr_x_CuO_4_. La_2−x_Sr_x_NiO_4_ shows only localized holes with Ni3d^8^L configuration, where the ligand hole L is a O(2*p*^5^) localized charge^23–25^. This system is known to show SDW as well as CDW at low temperature. The dynamics of the incommensurate SDW has been recently investigated by X-ray Photon Correlation Spectroscopy (XPCS)^20^. The spin-stripe order has been found to be spatially and temporally destabilized at low temperature, resembling the anomalous decay of the stripe order in cuprates, which is generally ascribed to the competing onset of superconductivity^1^. Since superconductivity is absent in this nickelate the atypical low temperature behavior has been explained with an energy barrier for thermally activated lattice fluctuations at nanoscale^21,22^.

In this work we have studied the spatial distribution of the CDW fluctuations rate in La_2−x_Sr_x_NiO_4+y_ by combining resonant soft XPCS with SµXRD. SµXRD and XPCS provide a visualization of the inhomogeneous landscape and the rate of fluctuations of CDW textures, respectively. Using XPCS we have found that the CDW fluctuations rate increases at low temperatures, as it occurs for the spin order^20^. This dynamic rate has been found to be correlated with the CDW coherence length. The spatial map of CDW correlation length has been reconstructed by SµXRD with micrometer resolution. The results show a phase separation between (i) *dynamic* CDW puddles with short coherence length and faster fluctuation rate and (ii) *quasi-static* CDW puddles with long coherence length and slower fluctuation rate. In this way, we have been able to visualize the spatially inhomogeneous dynamics of CDW in La_2−x_Sr_x_NiO_4_, which is a key step to shed light on the inhomogeneous spatial distribution of charge density wave puddles in complex quantum materials.

## Results and discussion

**Figure 1:**
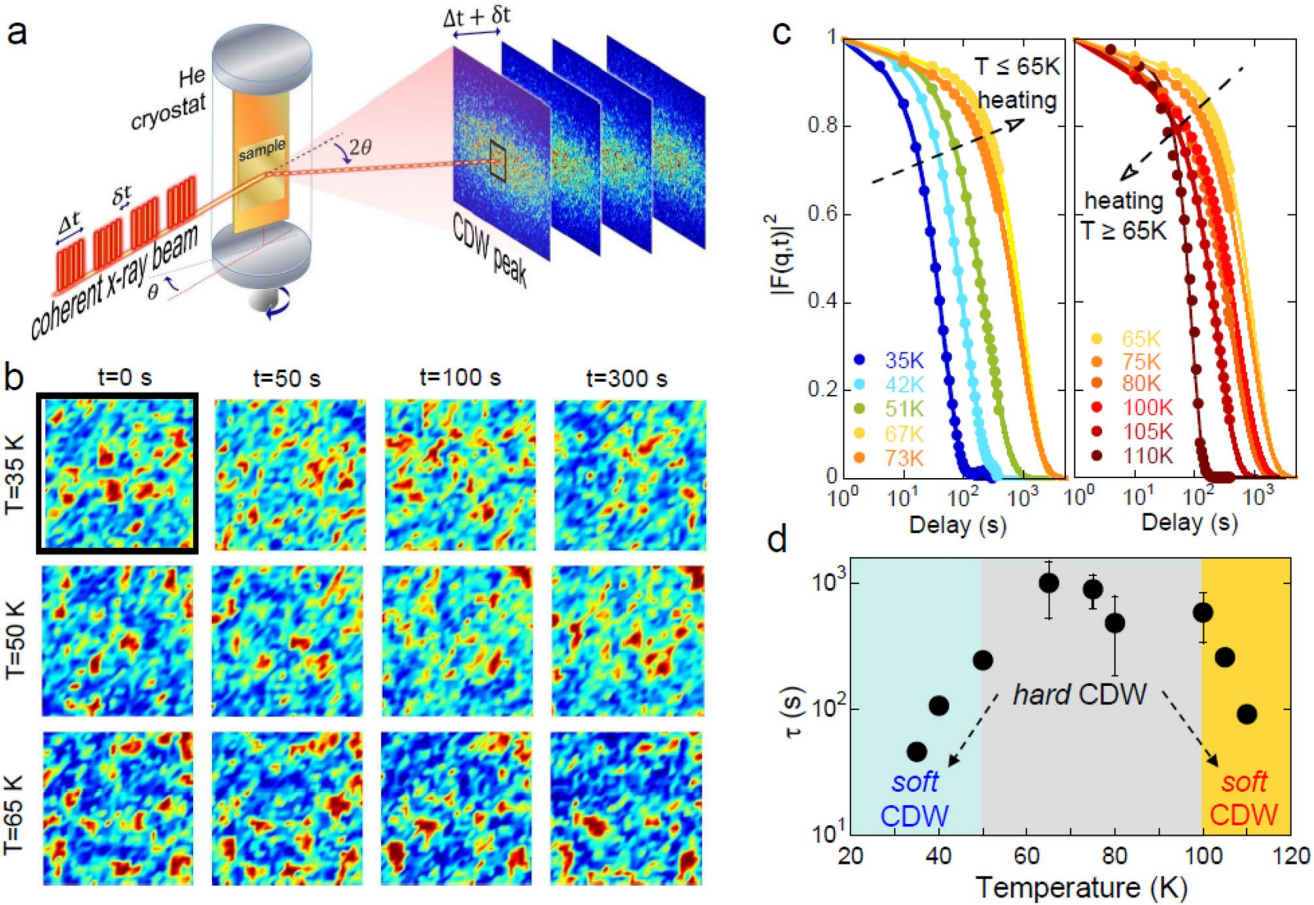
Temperature dependence of CDW dynamics measured by resonant soft X-ray Photon Correlation Spectroscopy in La_1.71_Sr_0.29_NiO_4_. (**a**) Experimental setup for the XPCS measurements. Coherent X rays hit the sample placed at the 2θ angle of the CDW peak. A time series of X-ray coherent diffraction images allows to study the development of the speckle spatial correlations. The region of interest, ROI, inside the square, where the intensity is more intense and stable has been used for analysis. (**b**) Images of speckle data recorded at T = 35 K, T = 50 K and T = 65 K in the selected ROI at different times. The larger the delay time, the more the speckle pattern differs from the initial one. (**c**) Autocorrelation function vs. delay measured at the indicated different temperatures. Experimental data are indicated by full circles along the solid lines fits obtained by using the stretched exponential model of Eq. (2). (**d**) Time delay, τ, extracted by fitting the |F(q,t)|^2^ using Eq. (2). The time delay of fluctuations reaches values higher by a factor 100 passing from the *hard* region to the *soft* regions at both high and low temperature.

In La_1−x_Sr_x_NiO_4_^15^ for 0.33 > x > 0.25 the CDW and SDW extend in real space diagonally to the Ni–O bond directions, along the orthorhombic unit cell. For samples with tetragonal symmetry, as the one studied here, the stripes order itself breaks the rotational symmetry of the *ab*-plane and therefore stripes with two different orientations show up related by a 90-degree rotation around *c*-axis. It has been found by neutron diffraction that incommensurate SDW at the lowest momentum transfer occur at wave vectors (1 − ε, 0, 0), where ε is a temperature dependent incommensurability value. CDW reflections can be detected separately in the k-space at (1 − q_h_, 0, L), where q_h_ = 2ε and L is odd. In this work we have investigated a La_1−x_Sr_x_NiO_4_ sample with a doping level of nominal Sr concentration x = 0.28, In previous X-ray diffraction studies on CDW in superconducting cuprates, it has been found that both the correlation length as well as the reflection intensity decrease as superconductivity arises for T < T_c_^1,10^. These results have been interpreted as evidence for competition between superconductivity and CDW order. However, a recent study^20^ on SDW order in the same insulating nickelate as studied here, shows that the SDW order decreases below 70 K while there is no onset of superconductivity. In this context, it is also important to know the behavior of the CDW, in particular at low temperatures. Thus, we have measured the low temperature time fluctuations of the CDW peak with wavevector **q**_**CDW**_ = (1 − 2ε)a* + c*, by using resonant XPCS, illuminating the sample by soft coherent X-rays with the X-ray energy tuned to the Ni 2*p* → 3*d* (L_3_) resonance. The sketched experimental layout is shown in Fig. 1a. XPCS measurements have been performed at the Advanced Light Source (ALS) at Lawrence Berkeley National Laboratory. We collected time series of coherent X ray diffraction images of the CDW peak at different temperatures (see Fig. 1b and “Methods”). Thanks to the coherence of the X-ray beam, the resonant CDW peak shows many speckles due to the disorder of the charge order domains in the coherently illuminated sample volume. The temporal evolution of the speckles reveals the dynamical behavior within the CDW order. To quantify this evolution, we have determined the intensity autocorrelation function g_2_ which leads to the modulus of the intermediate scattering function |F(q, t)| through

1 $$ g_{2} \left( t \right) = \frac{{\left\langle {I\left( \tau  \right)I\left( {\tau  + t} \right)} \right\rangle _{\tau } }}{{\left\langle {I\left( \tau  \right)} \right\rangle _{\tau }^{2} }} = 1 + A\left| {F\left( {q,t} \right)} \right|^{2}  $$

where <…>_τ_ denotes the integration over the whole set of frames recorded for one temperature. We restrict our analysis to the central part of the Bragg peak, indicated by the black square, where peak intensity is high and more stable. We report no significant indications for different temporal behavior in different regions of the peak. We collected several time-series in the temperature range from 35 to 105 K; the square of |F(q,t)| is shown for each of these temperatures in Fig. 1c. All curves show a characteristic exponential decay. We can distinguish two temperature ranges: in the first one (left panel), heating the sample up to 65 K, the characteristic decay time increases (i.e. the curves shift to the right), while in the second regime, for T > 65 K, the dynamic behavior is inverted and the characteristic decay time decreases, in agreement with previous works^14^. In order to quantify this behavior, we fitted the autocorrelation function by a stretched exponential Kohlrausch–Williams–Watts (KWW) model:

2 $$  \left| {F(t)} \right|^{2}  = e^{{ - (t/\tau )^{\beta } }} $$

where τ is the characteristic decay time of the dynamics, *β* is the so-called stretching exponent. The results of least-squares-fits to the data for τ are summarized in Fig. 1d. The value of β scatters around 1.1 (1.1 ± 0.1). For decreasing temperatures the decay time, τ grows down to 100 K in the yellow region, by more than a factor ten. Then τ stays fairly constant in the 50–100 K range (grey region) and decreases again below 50 K. Thus, we can clearly distinguish *quasi-static* (or ‘strongly pinned’) CDW puddles, namely hard CDW in the 50–100 K range from more *dynamical* (or ‘weakly pinned’) CDW puddles, namely soft CDW, at both higher (T > 100 K) and lower (T < 50 K) temperatures.

**Figure 2:**
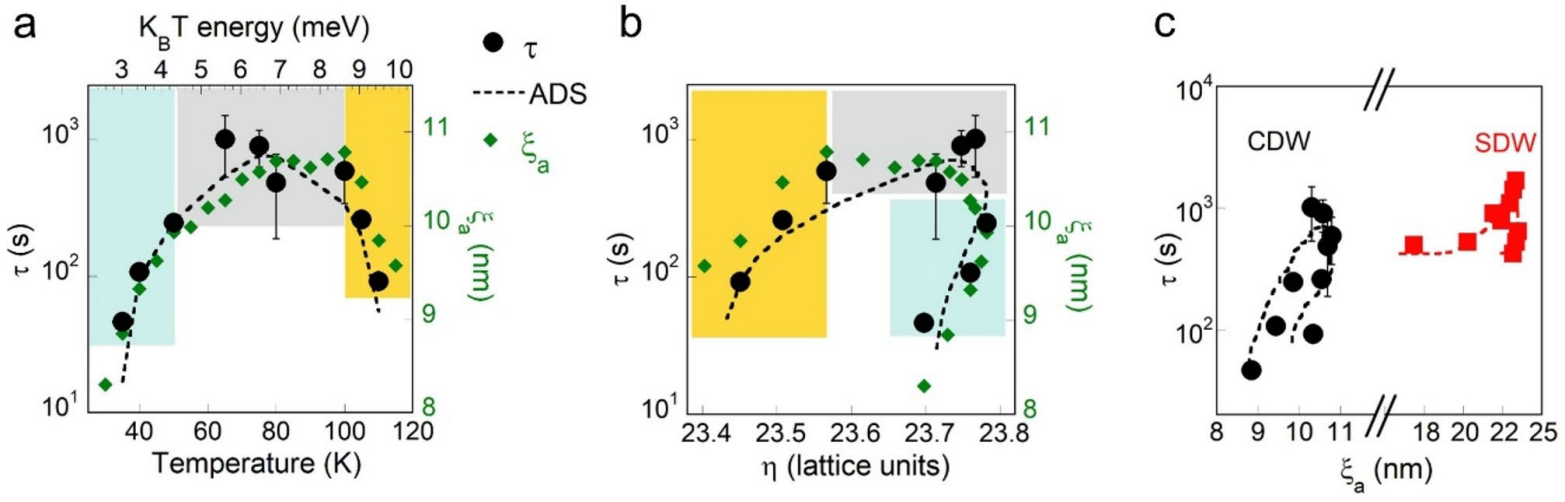
Correlations between CDW and SDW domain size and dynamics in La_1.71_Sr_0.29_NiO_4_. (**a**) Characteristic decay times, τ, of CDW (black full circles) along the fit (dashed line) extracted in the modified Activated Dynamical Scaling (ADS) model described by Eq. (3), as a function of temperature in logarithmic scale. The behavior of τ is compared with the (full diamonds) in plane coherence length, ξ_a_. We observe how lower delays correspond to lower coherence lengths that is to smaller domains in both low temperature (light blue) and high temperature (yellow) regions. (**b**) Time delay, τ, (black full circles), and ξ_a_ (full diamonds) of CDW, as a function of the incommensurability, η = 14/H, where H is the CDW wavevector along the a* direction (see “Methods”). Here lower values of delays and coherence lengths occur in the low temperature (light blue) and high temperature (yellow) regions. The fit of τ as a function of T and ξ_a_ extracted from the ADS theory is represented by the black dashed line. (**c**) The correspondence between time delays, τ, and domain size, ξ_a_, for CDW has been compared with time delays and domain size in SDW measured in the same sample, as reported in ref. ^20^. The dashed lines represent always the fit of τ as a function of T and ξ_a_ extracted from the ADS theory.

**Figure 3:**
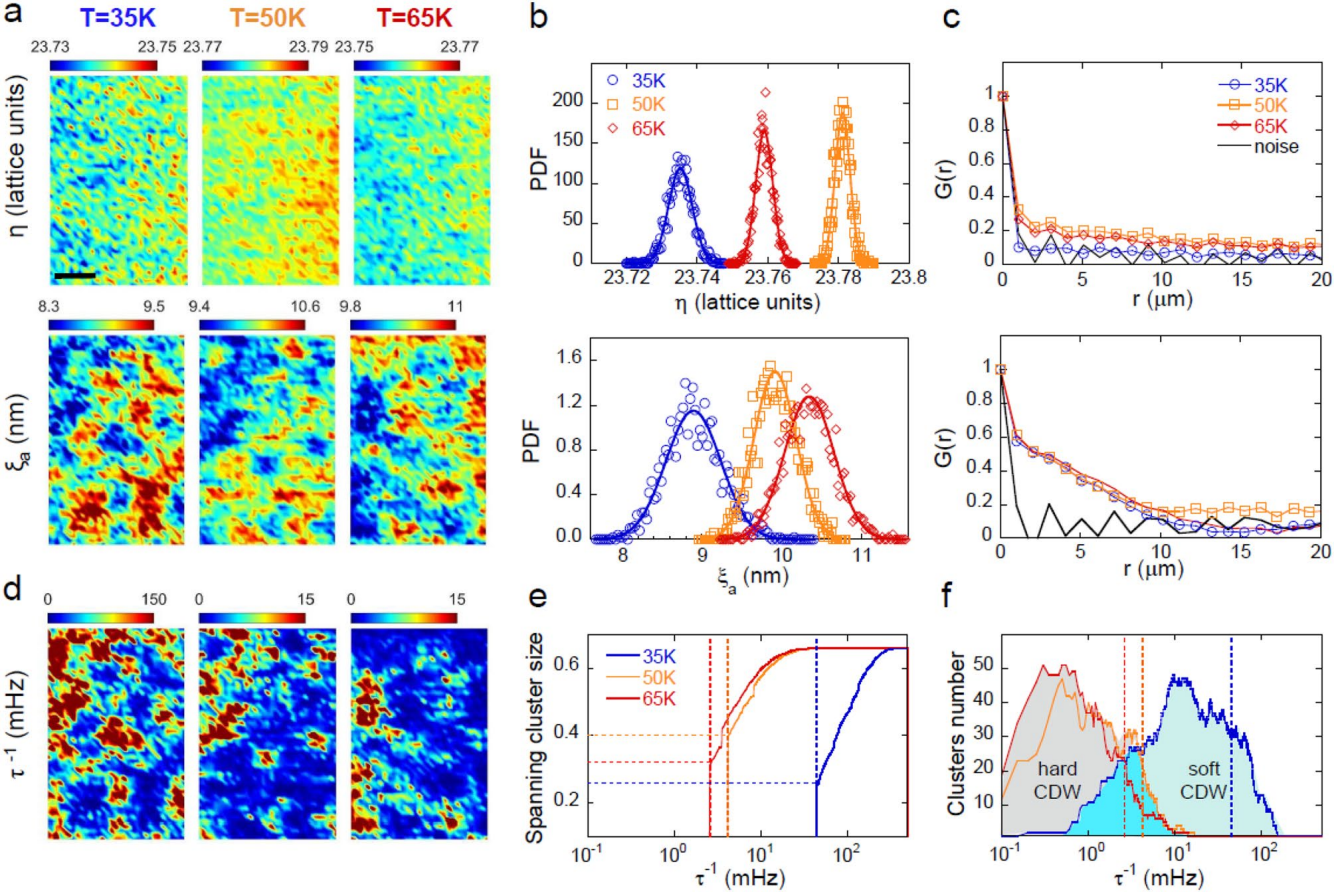
Mapping the small/large and fast dynamics CDW domains by X-ray micro diffraction in La_1.71_Sr_0.29_NiO_4_. (**a**) Maps (**b**) PDF and (**c**) G(r) of CDW peak incommensurability, η, in plane coherence length, ξ_a_, measured at T = 35 K, 50 K, and 65 K. Values of each single pixel have been obtained recording the CDW peak in a specific (x, y) position of the sample. In order to reconstruct the spatial maps, the sample has been scanned over an area of about 66 × 100 μm^2^ in steps of 2 μm in both directions. The scale bar shown in the upper frame corresponds to 20 μm. We can observe the narrow fluctuations of η that decays in few microns. On the other hand, ξ_a_ form puddles of about 10 μm of radius. (**d**) Maps of CDW peak frequency, τ^-1^, at T = 35 K, 50 K and 65 K. (**e**) Spanning clusters size and (**f**) forming clusters number calculated for the maps in (**a**). The percolation thresholds are represented by the vertical dashed lines.

In order to shed light on the correlation between the decay time, τ and the in-plane correlation length, ξ_a_ we have plotted τ and, ξ_a_, as a function of the temperature in Fig. 2a. The in-plane coherence length is given by ${\xi }_{a}=1/2\pi \Delta (H)$ where $\Delta (H)$ is the full width at half maximum of the CDW peak (see “Methods”). The data show that *soft* CDW (small τ) occur in the presence of shorter correlation lengths (small ξ_a_) at both low and high temperatures. It is known that the relation between the fluctuation times, temperature and correlation length can be described by the *activated dynamical scaling* (ADS) model^21,22^. In this model the competition between different interactions is controlled by the distribution of the energy barriers, which typically evolve as a power of the length scale which characterize the process^22^. The model is appropriate to describe the glassy freezing of charge and spin stripes below 50 K as shown by measurements of temperature dependent dielectric responses^31–34^. In the ADS model the fluctuation time obeys to τ ~ exp(Cξ^z^/T) where ξ is the correlation length, and z the so-called dynamical critical exponent, which is typically around 2 and C is a constant. The term Cξ^z^ times k_B_, the Boltzmann constant, is an effective energy barrier height for activated fluctuations. We have developed our computer code for the ADS model introducing a constant correlation length, ${\xi }_{a}^{0}$, to take structural defects of the sample into account. The dashed line in Fig. 3a shows the fitting curve to the measured characteristic decay times τ obtained by the modified ADS model

3 $$\tau ={\tau }_{0}{e}^{{C{({\xi }_{a}-{\xi }_{a}^{0})}^{z}}/{T}}+{\tau }_{1}$$

with ${\xi }_{a}^{0}$ = 7.56 nm, z = 2.2, and τ_1_ is an offset. The ADS model allows us to fit our experimental data fairly well in Fig. 2b where we plot the decay times, τ, and coherence lengths, ξ_a_, as a function of the incommensurability, η = 14/H, where H is the CDW wavevector along the a* direction (see “Methods”). One key result of this work is that the *soft* CDW signal with short coherence lengths occurs both with lower and higher incommensurability values. The CDW behaviour alongside the ADS model has been compared with SDW behaviour, described in^20^, in Fig. 2c. The full circles represent the experimental data, while the dashed lines represent the fit of τ as a function of ξ_a_ extracted from the ADS theory. The maximum values of τ and ξ appear at intermediate temperatures (50 K < T < 100 K), while we observe decreasing values of both τ and ξ_a_ at low (T < 50 K) and high (T > 100 K) temperature range, which provides compelling evidence of the non-monotonic temperature dependence. We observe that the maximum values of the decay time, τ of SDW is around two times the maximum values of τ of CDW. At the same time, also the coherence length ξ_a_ of SDW domains are around two times the coherence length of CDW domains. This shows that larger domains (larger coherence length) present slower dynamics (larger decay time). Therefore, the larger SDW puddles show slower fluctuations with larger characteristic decay times. The different coupling of spin and charge with lattice fluctuations could be at the origin of the different shape of τ(T) in SDW and CDW.

In order to unveil the differences between the spatial inhomogeneity of CDW in nickelates and the inhomogeneity observed in cuprates^10^, we used SµXRD to probe the local charge order via the mapping of the CDW superlattice peak. Above, we have shown that there are two types of CDW in the nickelates: fast fluctuating CDW puddles with short coherence length, *soft CDW*, and slower fluctuating CDW puddles with longer coherence length, *hard CDW*. Therefore, we investigated how these two types of CDW are distributed in space, focusing on the low temperature region where quantum fluctuations are expected for domain walls in incommensurate CDW.

We performed SµXRD measurements on the P10 beamline of the PETRA III synchrotron. The spatial distribution of the CDW peak incommensurability, η^19,35^ and the in-plane coherence length, ξ_a_, have been calculated as described in “Methods”, and visualized over areas of 66 × 100 μm^2^ in steps of 2 μm in both directions. Maps collected by scanning over the same sample area in the low temperature region at T = 30 K, T = 50 K and T = 65 K are shown in Fig. 3a. In the spatial maps, red (blue) areas correspond to a higher (lower) CDW peak incommensurability and coherence length. We clearly observe a different texture made of larger domains in the coherence length maps. Figure 3b shows a statistical analysis of the CDW incommensurability and coherence length spatial distribution in terms of the probability density function, PDF. At all temperatures, the incommensurability distributions follow a narrow normal distribution, attesting the good quality of our sample. The PDF are normally distributed but fluctuations of coherence length are larger over a factor 100, respect to the incommensurability fluctuations. This is confirmed by the spatial intensity correlation function, G(r) (Fig. 3c), where r = |**R**_**i**_ − **R**_**j**_| is the distance between x–y positions **R**_**k**_ on the sample, G(r) function shows that the spatial correlation of incommensurability decay quite fast inside the resolution limited distance of 2 µm, while the spatial correlations of coherence lengths decay slower, outside distances of r = 10 μm. Details on the G(r) calculations have been described elsewhere in^44^.

In Fig. 3d we show the reconstructed map for the decay rate, given by τ^−1^, calculated point by point using the ADS model of Eq. (3), at each temperature T = 35 K, 50 K and 65 K. The spatial inhomogeneity of the CDW rate fluctuations, τ^−1^, has been quantified by studying the connectivity in the maps of τ^−1^ using standard cluster 2D percolation analysis^50^. We consider two adjoining pixels to belong to the same cluster if they are connected along the horizontal, vertical, or diagonal direction and have rate below a threshold values (τ^−1^)*. We have calculated all the forming clusters, picking out the cluster with the largest extent, as a function of (τ^−1^)*. When we find a spanning cluster, with size equal to the system size, the system percolates. Thus in each map of τ^−1^, measured at the three different temperatures in the low temperature regime, we have calculated the percolation threshold, p(τ^−1^) the spanning cluster size and the number of clusters formed. As found in XPCS measurements, at T = 35 K and T = 65 K we got *soft* and *hard* CDW, respectively, given by the minimum and maximum value of time decay τ. The *soft* CDW with smaller coherence length, ξ_a_ and τ, show a largest percolation threshold of the fluctuations rate, τ^−1^. As CDW become *hard*, with larger coherence length ξ_a_ and τ, the percolation threshold of the τ^−1^ rate fluctuations decreases, as shown in Fig. 3e. In addition, in the *hard* regime, we find a larger number of forming clusters at low fluctuations rate, τ^−1^, as reported in Fig. 3f. Thus, this cluster analysis provides a further criterion to characterize the *soft* and *hard* CDW dynamics.

Summarizing, we can say that in the *soft* regime, at T < 50 K and T > 100 K, we observe CDW with smaller coherence length, ξ_a_, and faster dynamics (smaller time, τ) forming clusters at higher fluctuations rate, τ^−1^ and percolating at τ^−1^ = 44.5 mHz. On the other hand, in the *hard* regime, at 50 K < T < 100 K, we observe CDW with larger coherence length, ξ_a_ and slower dynamics (larger time decay, τ) forming clusters at lower fluctuations rate, τ^−1^, and percolating at τ^−1^ = 2.5 mHz. At T = 50 K we get a case where CDW values of τ and ξ_a_ are in between those found in the *soft* and *hard* regimes; here CDW percolate at fluctuations rate values of τ^−1^ = 4.2 mHz falling in between the percolation thresholds of *soft* and *hard* regimes.

## Conclusions

We have investigated the spatial distribution of the charge density wave fluctuations rate in La_1.72_Sr_0.28_NiO_4_ in the low temperature regime for T < 65 K. We have combined scanning micro X-ray diffraction (SµXRD) with resonant soft X-ray photon correlation spectroscopy (XPCS) to get spatial and temporal correlation landscapes of CDW textures. We report here clear evidence for CDW motion in nickelates in contrast with CDW remarkable stability in cuprates. The CDW fluctuations rate in nickelates increases with decreasing CDW coherence length in agreement with spin order dynamics^20^. We have found the anomalous drop at low temperatures of the CDW coherence length with the increasing fluctuation rate. We identify soft CDW puddles characterized by smaller size and higher mobility and hard CDW puddles with larger size and low mobility. Cluster analysis of SµXRD spatial maps of coherence lengths shows a phase separation between percolating soft CDW dynamic puddles and hard CDW puddles. The visualization of both spatial and time inhomogeneous landscape of charge density wave in La_2−x_Sr_x_NiO_4_ provides a novel nanoscale phase separation phenomenon in complex quantum materials, supporting the proposals that this nanoscale phase separation involves lattice degrees of freedom.

In summary, (i) the inhomogeneous spatial distribution of CDW in LSNO, (ii) the unusual non-monotonic temperature dependence of spatial and temporal CDW correlation and (iii) soft CDW signal with short coherence lengths occurring both with lower and higher incommensurability values, provide key information for the search of novel topological states at nanoscale in quantum complex matter ^51,52^. The nanoscale topology determines quantum functionality beyond traditional paradigms of Fermi liquid theory and spontaneous symmetry breaking in homogeneous quantum materials.

## Methods

Single-crystalline La_1.72_Sr_0.28_NiO_4_ was grown by floating zone technique. The seed and feed rods were prepared from polycrystalline powder obtained by solid state reaction of La_2_O_3_, SrCO_3_ with an excess of NiO. The reaction was performed at 1200 °C for 20 h with intermediate grinding. The rods were densified at 1500 °C for 5 h. The synthesis were carried out in air.

The temperature dependent scanning micro X-ray diffraction μXRD experiments were carried out at the Coherence Beamline P10 of PETRA III synchrotron Hamburg. The synchrotron radiation source was a 5 m long undulator (U29). The x-ray beam was monochromatized by a cooled Si(111) double-crystal monochromator with a bandwidth of ΔE/E ∼ 1.4 × 10^–4^. The collimated coherent x-ray beam was focused using a beryllium refractive lens (CRL) transfocator to a size of about 2 × 2.5 μm^2^ at the sample positioned ~ 1.6 m downstream of the transfocator center. The incident flux on the sample was about 10^11^ photons/s. The windows of the He cryostat as well as the entrance window of the evacuated detector flight path were covered by 25 μm thick Kapton foils. The incident photon energy was set slightly below the Ni *K*-edge to minimize a possible fluorescence background. The scattered signal was detected using the large horizontal scattering setup with a sample-to-detector distance of 5 m. A PILATUS 300 K detector was used to record the x rays scattered by the sample. For the measurements, the sample was cooled to the lowest temperature 30 K and the measurements were performed during a heating cycle. We aligned the crystal to detect the charge ordering satellite of the 100 reflection, which appears below T_ξ_ = 120 K. The incommensurate charge density wave satellite appears at q_h_ = 2ε = 0.59. The scanning maps shown in Fig. 3 have been obtained by translating the sample in steps of 2 μm in both directions. We have mapped the spatial distribution of the (q_h_,0,1) CDW peak over areas of 100 × 66 μm^2^.

The CDW incommensurability is measured by the lattice units of the superstructure η related to its period, 1/H. The incommensurate CDW with 0.25 < ε < 0.33 consists of the mixture of the ε = 1/n and the ε = 1/m order, i.e. alternating the so-called n = 3 stripes portions and m = 4 stripes portions. Indeed, quasi-commensurate periods $\uplambda =1/\varepsilon = \frac{n x+my }{n+m}$ intermediate between two main commensurate wave-vectors 1/n and 1/m with periods of m and n lattice units, respectively, occur at integer numbers of lattice unit cells $\eta =\frac{n+m}{\varepsilon }$. Each quasi-commensurate phase (QCP) corresponds to a modulation wave locked with the underline lattice onto a rational number. This sequence of quasi-commensurate phases is called the Devil's staircase^17–19^. Choosing n = 2 × 3 and m = 2 × 4 the incommensurability in our sample is given by η = 14/H which approaches the quasi-commensurate phase with η = 24 lattice units around T = 65 K.

The CDW coherence length has been extracted from the full widths at half maxima, $\Delta (H)$, $\Delta (L)$ by fitting the profiles along H and L directions with Lorentzian-squared line shapes. In particular, the coherence length along the **a** (in-plane) and **c** (out of plane) crystallographic directions have been calculated as ${\xi }_{a}=1/2\pi \Delta (H)$ and ${\xi }_{c}=1/2\pi \Delta (L)$. The three temperatures chosen in the SµXRD measurements have been selected in order to map CDW in the two different dynamical regimes: soft (T = 35 K) and hard (T = 65 K), while at 50 K we study the intermediate case.

X-ray photon correlation spectroscopy (XPCS) measurements were carried out at beamline 12.0.2 of the Advanced Light Source at Lawrence Berkeley National Laboratory (USA). The experiment was conducted in a θ-2θ reflection geometry. Tuning the energy of the incoming linear σ polarized x-ray to the L_3_-edge of Ni (852 eV) brings about magnetic sensitivity. The transverse coherence of the beam was established by placing a 5 microns pinhole approximately 3 mm in front of the sample. A CCD placed 0.45 m away served as detector. The temperature range for XPCS has been chosen similar to that of^20^, in order to compare the data for SDW and CDW dynamics.

## Data Availability

All data generated or analyzed during this study are included in this published article. Further information on the current study are available from the corresponding author on reasonable request.

## References

[1] Chang J, Blackburn E, Holmes AT, Christensen NB, Larsen J, Mesot J (2012). Direct observation of competition between superconductivity and charge density wave order in YBa_2_Cu_3_O_6.67_. Nat. Phys..

[2] Caprara S, Di Castro C, Seibold G, Grilli M (2017). Dynamical charge density waves rule the phase diagram of cuprates. Phys. Rev. B.

[3] Peng YY, Fumagalli R, Ding Y, Minola M, Caprara S, Betto D (2018). Re-entrant charge order in overdoped (Bi, Pb) 212S_1.88_CuO_6+__δ_ outside the pseudogap regime. Nat. Mater..

[4] Uchida SI (2021). Ubiquitous Charge Order Correlations in High-Temperature Superconducting Cuprates. J. Phys. Soc. Jpn..

[5] Forgan EM, Blackburn E, Holmes AT, Briffa AKR, Chang J, Bouchenoire L (2015). The microscopic structure of charge density waves in underdoped YBa_2_ Cu_3_O_6.54_ revealed by X-ray diffraction. Nat. Commun..

[6] Peng YY, Husain AA, Mitrano M, Sun SL, Johnson TA, Zakrzewski AV (2020). Enhanced electron-phonon coupling for charge-density-wave formation in La_1.8−x_Eu_0.2_Srx CuO_4+__δ_. Phys. Rev. Lett..

[7] Arpaia R, Caprara S, Fumagalli R, De Vecchi G, Peng YY, Andersson E (2019). Dynamical charge density fluctuations pervading the phase diagram of a Cu-based high-Tc superconductor. Science.

[8] Bianconi A (1994). On the Fermi liquid coupled with a generalized Wigner polaronic CDW giving high Tc superconductivity. Solid State Commun..

[9] Bianconi A (2013). Shape resonances in superstripes. Nat. Phys..

[10] Campi G, Bianconi A, Poccia N, Bianconi G, Barba L, Arrighetti G (2015). Inhomogeneity of charge-density-wave order and quenched disorder in a high-T_c_ superconductor. Nature.

[11] Chen XM, Thampy V, Mazzoli C, Barbour AM, Miao H, Gu GD (2016). Remarkable stability of charge density wave order in La_1.875_ Ba_0.125_ CuO_4_. Phys. Rev. Lett..

[12] Chen XM, Mazzoli C, Cao Y, Thampy V, Barbour AM, Hu W (2019). Charge density wave memory in a cuprate superconductor. Nat. Commun..

[13] Miao H, Fabbris G, Koch RJ (2021). Charge density waves in cuprate superconductors beyond the critical doping. NPJ Quant. Mater..

[14] Shen Y, Fabbris G, Miao H, Cao Y, Meyers D, Mazzone DG (2021). Charge condensation and lattice coupling drives stripe formation in nickelates. Phys. Rev. Lett..

[15] Ulbrich H, Braden M (2012). Neutron scattering studies on stripe phases in non-cuprate materials. Physica C.

[16] Katsufuji T, Tanabe T, Ishikawa T, Yamanouchi S, Tokura Y, Kakeshita T, Kajimoto R, Yoshizawa H (1999). Commensurability effect on the charge ordering of La_2−x_Sr_x_NiO_4_. Phys. Rev. B.

[17] Yoshizawa H, Kakeshita T, Kajimoto R, Tanabe T, Katsufuji T, Tokura Y (2000). Stripe order at low temperatures in La_2−x_Sr_x_NiO_4_ with 0.289≲x≲0.5. Phys. Rev. B.

[18] Lee S-H, Cheong S-W, Yamada K, Majkrzak CF (2001). Charge, canted spin order in La_2−x_Sr_x_NiO_4_ (x = 0.275, 1/3). Phys. Rev. B.

[19] Bak P (1982). Commensurate phases, incommensurate phases and the devil's staircase. Rep. Prog. Phys..

[20] Ricci A, Poccia N, Campi G, Mishra S, Müller L, Joseph B (2021). Measurement of spin dynamics in a layered nickelate using X-ray Photon correlation spectroscopy: Evidence for Intrinsic destabilization of Incommensurate stripes at low temperatures. Phys. Rev. Lett..

[21] Karmakar S, Dasgupta C, Sastry S (2014). Growing length scales and their relation to timescales in glass forming liquids. Annu. Rev. Condens. Matter Phys..

[22] Fisher DS (1987). Activated dynamic scaling in disordered systems (invited). J. Appl. Phys..

[23] Zaanen J, Littlewood PB (1994). Freezing electronic correlations by polaronic instabilities in doped La_2_NiO_4_. Phys. Rev. B.

[24] Raczkowski M, Frésard R, Oleś AM (2006). Microscopic origin of diagonal stripe phases in doped nickelates. Phys. Rev. B.

[25] Rościszewski K, Oleś AM (2011). Jahn-Teller mechanism of stripe formation in doped layered La_2−x_Sr_x_NiO_4_ nickelates. J. Phys.: Condens. Matter.

[26] Schüßler-Langeheine C (2005). Spectroscopy of stripe order in La_1.8_Sr_0.2_NiO_4_ Using Resonant Soft X-Ray Diffraction. Phys. Rev. Lett..

[27] Lu Y, Frano A, Bluschke M, Hepting M, Macke S, Strempfer J, Wochner P, Cristiani G, Logvenov G, Habermeier HU, Haverkort MW, Keimer B, Benckiser E (2016). Quantitative determination of bond order and lattice distortions in nickel oxide heterostructures by resonant x-ray scattering. Phys. Rev. B.

[28] Campi G, Poccia N, Joseph B, Bianconi A, Mishra S, Lee J (2019). A direct visualization of spatial inhomogeneity of spin stripes order in La_1.72_ Sr_0.28_ NiO_4_. Condens. Matter.

[29] Klingeler R, Büchner B, Cheong SW, Hücker M (2005). Weak ferromagnetic spin and charge stripe order in La_5∕3_Sr_1∕3_NiO_4_. Phys. Rev. B.

[30] Merritt AM, Reznik D, Garlea VO, Gu GD, Tranquada JM (2019). Nature and impact of stripe freezing in La_1.67_Sr_0.33_NiO_4_. Phys. Rev. B.

[31] Park T, Nussinov Z, Hazzard KRA, Sidorov VA, Balatsky AV, Sarrao JL, Cheong S-W, Hundley MF, Lee J-S, Jia QX, Thompson JD (2005). Novel dielectric anomaly in the hole-doped La_2_Cu_1__−__x_Li_x_O_4_ and La_2x_Sr_x_NiO_4_ insulators: Signature of an electronic glassy state. Phys. Rev. Lett..

[32] Filippi M, Kundys B, Agrestini S, Prellier W, Oyanagi H, Saini NL (2009). Charge order, dielectric response, and local structure of La_5/3_Sr_1/3_NiO_4_ system. J. Appl. Phys..

[33] Liu XQ, Jia BW, Yang WZ, Cheng JP, Chen XM (2010). Dielectric relaxation and polaronic hopping in Al-substituted Sm_1.5_Sr_0.5_NiO_4_ ceramics. J. Phys. D.

[34] Petersen J, Bechstedt F, Furthmüller J, Scolfaro LM (2018). Spontaneous symmetry breaking and electronic and dielectric properties in commensurate La7/4Sr1/4CuO_4_ and La5/3Sr1/3NiO4. Phys. Rev. B.

[35] Campi G, Bianconi A, Ricci A (2021). Nanoscale phase separation of incommensurate and quasi-commensurate spin stripes in low temperature spin glass of La_2− x_ Sr_x_NiO_4_. Condens. Matter.

[36] Kagan MY, Kugel KI, Rakhmanov AL (2021). Electronic phase separation: Recent progress in the old problem. Phys. Rep..

[37] Bianconi A, Poccia N, Sboychakov AO, Rakhmanov AL, Kugel KI (2015). Intrinsic arrested nanoscale phase separation near a topological Lifshitz transition in strongly correlated two-band metals. Supercond. Sci. Technol..

[38] Kresin V, Ovchinnikov Y, Wolf S (2006). superconductivity and Inhomogeneous the “pseudogap” state of novel superconductors. Phys. Rep..

[39] Dagotto E, Hotta T, Moreo A (2001). Colossal magnetoresistant materials: The key role of phase separation. Phys. Rep..

[40] Zaanen J (2010). High-temperature superconductivity: The benefit of fractal dirt. Nature.

[41] Agrestini S, Metallo C, Filippi M, Simonelli L, Campi G, Sanipoli C (2004). Substitution of Sc for Mg in MgB_2_: Effects on transition temperature and Kohn anomaly. Phys. Rev. B.

[42] Ricci A, Poccia N, Campi G, Joseph B, Arrighetti G, Barba L (2011). Nanoscale phase separation in the iron chalcogenide superconductor K_0.8_Fe_1.6_Se_2_ as seen via scanning nanofocused X-ray diffraction. Phys. Rev. B.

[43] Ricci A, Poccia N, Joseph B, Innocenti D, Campi G, Zozulya A (2015). Direct observation of nanoscale interface phase in the superconducting chalcogenide K_x_Fe_2−y_Se _2_ with intrinsic phase separation. Phys. Rev. B.

[44] Fratini M, Poccia N, Ricci A, Campi G, Burghammer M, Aeppli G, Bianconi A (2010). Scale-free structural organization of oxygen interstitials in La_2_CuO_4+ y_. Nature.

[45] Poccia N (2012). Optimum inhomogeneity of local lattice distortions in La_2_CuO_4+y_. Proc. Natl. Acad. Sci. USA.

[46] Campi G, Ricci A, Poccia N, Fratini M, Bianconi A (2017). X-rays writing/reading of charge density waves in the CuO_2_ plane of a simple cuprate superconductor. Condens. Matter Condens. Matter.

[47] Ricci A, Poccia N, Campi G, Coneri F, Caporale AS, Innocenti D (2013). Multiscale distribution of oxygen puddles in 1/8 doped YBa_2_Cu_3_O_6.67_. Sci. Rep..

[48] Campi G, Ricci A, Poccia N, Barba L, Arrighetti G, Burghammer M (2013). Scanning micro-X-ray diffraction unveils the distribution of oxygen chain nanoscale puddles in YBa_2_Cu_3_O_6.33_. Phys. Rev. B.

[49] Carlson EW (2015). Charge topology in superconductors. Nature.

[50] Stauffer D, Aharony A (1994). Introduction to Percolation Theory.

[51] Haldane FDM (2017). Nobel lecture: Topological quantum matter. Rev. Mod. Phys..

[52] Dagotto E, Burgy J, Moreo A (2003). Nanoscale phase separation in colossal magnetoresistance materials: Lessons for the cuprates?. Solid State Commun..

